# Benzyl 2-{4-[2-(4-chloro­benzoyl­amino)­eth­yl]phen­oxy}-2-methyl­propionate

**DOI:** 10.1107/S1600536811039249

**Published:** 2011-09-30

**Authors:** Ghulam Mustafa, Sania Tasneem, Islam Ullah Khan, Muhammad Ashfaq, Muhammad Nadeem Arshad

**Affiliations:** a Department of Chemistry, University of Gujrat, Gujrat, Pakistan; b Materials Chemistry Laboratory, Department of Chemistry, GC University, Lahore 54000, Pakistan; c X-ray Diffraction and Crystallography Laboratory, Department of Physics, School of Physical Sciences, University of the Punjab, Quaid-e-Azam Campus, Lahore 54590, Pakistan

## Abstract

In the title compound, C_26_H_26_ClNO_4_, the central phenyl­ene ring is oriented at dihedral angles of 5.06 (14) and 64.14 (5)°, respectively, with respect to aromatic rings of the benzyl and chloro­phenyl groups. The centroid–centroid distance between the central phenyl­ene ring and the aromatic ring of the benzyl group is 4.028 (12) Å. In the crystal, inter­molecular N—H⋯O hydrogen bond generate a chain along (100). C—H⋯O inter­actions are also observed.

## Related literature

For background to the drug bezafibrate [systematic name: 2-(4-{2-[(4-chloro­benzo­yl)amino]­eth­yl}phen­oxy)-2-methyl­prop­anoic acid], commonly used against hyperlipidemia, which has been found to decrease mRNA levels in adipocyte markers and increase fatty acid oxidation in primary cultures of adipocytes, see: Cabrero *et al.* (2001 ▶).
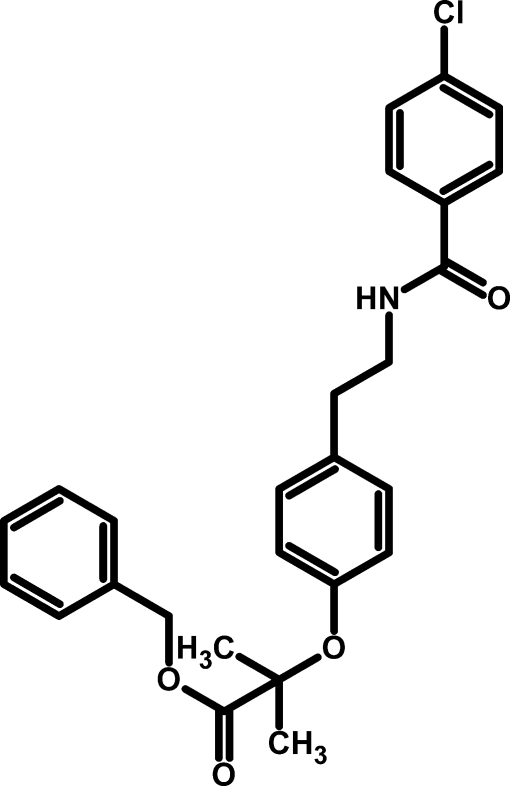


## Experimental

### 

#### Crystal data

C_26_H_26_ClNO_4_
*M* *_r_* = 451.93
Triclinic, 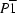

*a* = 5.5480 (1) Å
*b* = 11.0716 (3) Å
*c* = 18.9641 (5) Å
α = 82.247 (1)°
β = 86.915 (1)°
γ = 85.674 (1)°
*V* = 1149.81 (5) Å^3^
*Z* = 2
Mo *K*α radiation
μ = 0.20 mm^−1^
*T* = 296 K
0.41 × 0.19 × 0.13 mm

#### Data collection

Bruker Kappa APEXII CCD diffractometer
Absorption correction: multi-scan (*SADABS*; Bruker, 2007 ▶) *T*_min_ = 0.923, *T*_max_ = 0.975
26646 measured reflections
5750 independent reflections
4355 reflections with *I* > 2σ(*I*)
*R*_int_ = 0.019

#### Refinement

*R*[*F* ^2^ > 2σ(*F* ^2^)] = 0.049
*wR*(*F* ^2^) = 0.142
*S* = 1.05
5745 reflections
294 parameters
H atoms treated by a mixture of independent and constrained refinement
Δρ_max_ = 0.23 e Å^−3^
Δρ_min_ = −0.30 e Å^−3^

### 

Data collection: *APEX2* (Bruker, 2007 ▶); cell refinement: *SAINT* (Bruker, 2007 ▶); data reduction: *SAINT*; program(s) used to solve structure: *SHELXS97* (Sheldrick, 2008 ▶); program(s) used to refine structure: *SHELXL97* (Sheldrick, 2008 ▶); molecular graphics: *PLATON* (Spek, 2009 ▶); software used to prepare material for publication: *WinGX* (Farrugia, 1999 ▶) and *PLATON* (Spek, 2009 ▶).

## Supplementary Material

Crystal structure: contains datablock(s) I, global. DOI: 10.1107/S1600536811039249/ng5234sup1.cif

Structure factors: contains datablock(s) I. DOI: 10.1107/S1600536811039249/ng5234Isup2.hkl

Supplementary material file. DOI: 10.1107/S1600536811039249/ng5234Isup3.cml

Additional supplementary materials:  crystallographic information; 3D view; checkCIF report

## Figures and Tables

**Table 1:** Hydrogen-bond geometry (Å, °)

*D*—H⋯*A*	*D*—H	H⋯*A*	*D*⋯*A*	*D*—H⋯*A*
N1—H1*N*⋯O4^i^	0.848 (19)	2.540 (19)	3.350 (2)	160.3 (18)
C17—H17*B*⋯O4^ii^	0.97	2.57	3.532 (3)	172
C7—H7*B*⋯O2^i^	0.97	2.59	3.398 (3)	141

Symmetry codes: (i) 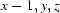; (ii) 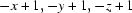.

**Fig. 1.**
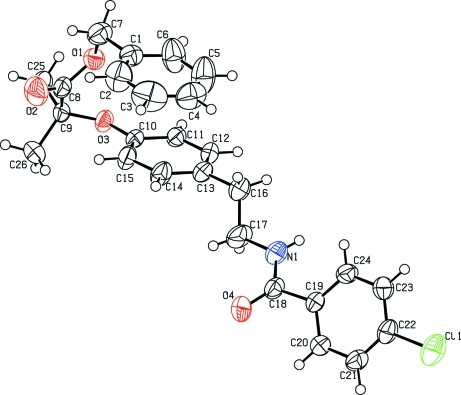
The ORTEP diagram of (I) showing the thermal ellipsoids drawn at 50% probability level.

**Fig. 2.**
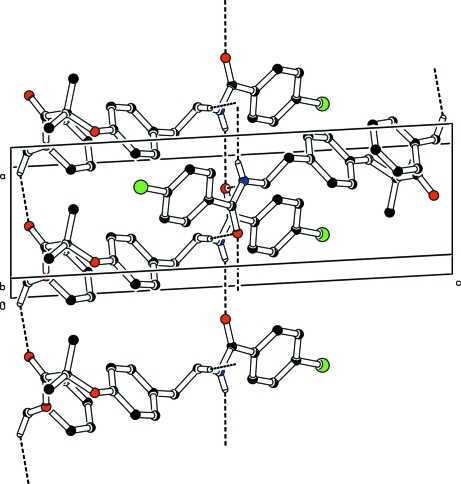
Unit cell diagram showing the N—H···O & C—H···O type interactions using dashed lines.

## supplementary crystallographic information

### Comment

Among the fibrate family of drugs bezafibrate (2-(4-{2-[(4-chlorobenzoyl)amino]ethyl}phenoxy)-2-methylpropanoic acid) is well known for its use against hyperlipidemia. The drug has also found effective in decreases of mRNA levels in adipocyte markers and increases fatty acid oxidation in primary culture of adipocytes (Cabrero *et al.*, 2001). Here in, we report the crystal structure of benzyl ester of bezafibrate (I).

The crysral structure of title compound consist of three aromatic rings. The aromatic ring (C1—C6) is oriented at dihedral angle of 5.06 (14)° with respect to other ring (C10—C15) and the centroid distance between these two rings is 4.028 Å. The chloro benzene ring (C19—C24) is twisted at dihedral angle of 64.14 (5)° with respect to the ring (C10—C15). The molecule is connected through only intermolecular hydrogen bonding of N—H···O and C—H···O type and generate an infinite chain along base vector (1 0 0).

### Experimental

A weighed amount of bezafibrate (0.40 g, 0.001105 moles) was dissolved in DMF (10 cm^3^) taken in a 100 ml conical flask. Then sodium hydride (0.0530 g; 0.002210 moles) washed with n-hexane was added in reaction flask. The reaction mixture was stirred for about 1 hr at an ambient temperature until the NaH disappeared. An equivalent amount of benzyl chloride (0.14 g, 0.001105 moles) was then added in the reaction mixture and stirred until the solution became clear. The reaction was monitored after regular intervals by TLC. After the consumption of benzyl chloride, the reaction mixture was poured over the crushed ice. The crude precipitates were filtered, washed with distilled water and crystallized with methanol to get colorless crystals. Melting point of product was noted as 374K.

### Refinement

All the C—H and H-atoms were positioned with idealized geometry with C_aromatic_—H = 0.93 Å, C_methylene_—H = 0.97 Å & C_methyl_—H = 0.96 Å and were refined using a riding model with *U*_iso_(H) = 1.2 *U*_eq_(C) for aromatic & methylene similarly *U*_iso_(H) = 1.5 *U*_eq_(C) for methyl carbon atoms. The N—H H-atom was refined via difference map. The reflections (0 0 1), (0 -1 1), (0 1 1), (0 1 0) and (0 0 2) have been omitted in final refinement.

### Crystal data

**Table:**

C_26_H_26_ClNO_4_	*Z* = 2
*M**_r_* = 451.93	*F*(000) = 476
Triclinic, *P*1	*D*_x_ = 1.305 Mg m^−^^3^
Hall symbol: -P 1	Melting point: 374 K
*a* = 5.5480 (1) Å	Mo *K*α radiation, λ = 0.71073 Å
*b* = 11.0716 (3) Å	Cell parameters from 9981 reflections
*c* = 18.9641 (5) Å	θ = 2.3–28.3°
α = 82.247 (1)°	µ = 0.20 mm^−^^1^
β = 86.915 (1)°	*T* = 296 K
γ = 85.674 (1)°	Needle, colorless
*V* = 1149.81 (5) Å^3^	0.41 × 0.19 × 0.13 mm

### Data collection

**Table:**

Bruker Kappa APEXII CCD diffractometer	5750 independent reflections
Radiation source: fine-focus sealed tube	4355 reflections with *I* > 2σ(*I*)
graphite	*R*_int_ = 0.019
φ and ω scans	θ_max_ = 28.4°, θ_min_ = 1.1°
Absorption correction: multi-scan (*SADABS*; Bruker, 2007)	*h* = −7→7
*T*_min_ = 0.923, *T*_max_ = 0.975	*k* = −14→14
26646 measured reflections	*l* = −25→25

### Refinement

**Table:**

Refinement on *F*^2^	Primary atom site location: structure-invariant direct methods
Least-squares matrix: full	Secondary atom site location: difference Fourier map
*R*[*F*^2^ > 2σ(*F*^2^)] = 0.049	Hydrogen site location: inferred from neighbouring sites
*wR*(*F*^2^) = 0.142	H atoms treated by a mixture of independent and constrained refinement
*S* = 1.05	*w* = 1/[σ^2^(*F*_o_^2^) + (0.061*P*)^2^ + 0.3442*P*] where *P* = (*F*_o_^2^ + 2*F*_c_^2^)/3
5745 reflections	(Δ/σ)_max_ = 0.001
294 parameters	Δρ_max_ = 0.23 e Å^−^^3^
0 restraints	Δρ_min_ = −0.30 e Å^−^^3^

### Special details

**Table:**

Geometry. All s.u.'s (except the s.u. in the dihedral angle between two l.s. planes) are estimated using the full covariance matrix. The cell s.u.'s are taken into account individually in the estimation of s.u.'s in distances, angles and torsion angles; correlations between s.u.'s in cell parameters are only used when they are defined by crystal symmetry. An approximate (isotropic) treatment of cell s.u.'s is used for estimating s.u.'s involving l.s. planes.
Refinement. Refinement of F^2^ against ALL reflections. The weighted R-factor wR and goodness of fit S are based on F^2^, conventional R-factors R are based on F, with F set to zero for negative F^2^. The threshold expression of F^2^ > 2σ(F^2^) is used only for calculating R-factors(gt) etc. and is not relevant to the choice of reflections for refinement. R-factors based on F^2^ are statistically about twice as large as those based on F, and R- factors based on ALL data will be even larger.

### Fractional atomic coordinates and isotropic or equivalent isotropic displacement parameters (Å^2^)

**Table:**

	*x*	*y*	*z*	*U*_iso_*/*U*_eq_	
Cl1	0.38362 (13)	−0.18807 (5)	0.70547 (3)	0.0904 (2)	
O3	0.10285 (18)	0.90785 (9)	0.18989 (5)	0.0445 (3)	
O1	0.0350 (2)	0.78925 (10)	0.07992 (6)	0.0496 (3)	
C10	0.1251 (2)	0.79595 (13)	0.23181 (7)	0.0384 (3)	
C19	0.4577 (3)	0.15357 (13)	0.55164 (7)	0.0396 (3)	
C11	−0.0609 (3)	0.77345 (14)	0.28206 (8)	0.0421 (3)	
H11	−0.1878	0.8324	0.2860	0.051*	
C22	0.4136 (3)	−0.05599 (14)	0.64589 (9)	0.0499 (4)	
C13	0.1266 (3)	0.57405 (15)	0.32228 (8)	0.0487 (4)	
C20	0.6232 (3)	0.12007 (15)	0.60402 (8)	0.0478 (4)	
H20	0.7508	0.1687	0.6072	0.057*	
C8	0.2515 (3)	0.83311 (14)	0.07877 (8)	0.0463 (3)	
C24	0.2680 (3)	0.08072 (14)	0.54764 (8)	0.0476 (4)	
H24	0.1544	0.1033	0.5131	0.057*	
C12	−0.0594 (3)	0.66391 (15)	0.32648 (8)	0.0482 (4)	
H12	−0.1862	0.6501	0.3600	0.058*	
C9	0.2523 (3)	0.93371 (14)	0.12628 (8)	0.0437 (3)	
C23	0.2462 (3)	−0.02508 (15)	0.59448 (9)	0.0512 (4)	
H23	0.1202	−0.0747	0.5913	0.061*	
N1	0.3016 (3)	0.32670 (14)	0.47282 (8)	0.0598 (4)	
C14	0.3122 (3)	0.59869 (16)	0.27238 (9)	0.0556 (4)	
H14	0.4398	0.5401	0.2688	0.067*	
O2	0.4214 (2)	0.80229 (15)	0.04190 (8)	0.0763 (4)	
C21	0.6010 (3)	0.01526 (16)	0.65165 (9)	0.0541 (4)	
H21	0.7116	−0.0066	0.6871	0.065*	
C16	0.1268 (4)	0.45288 (17)	0.37026 (10)	0.0617 (5)	
H16A	−0.0309	0.4454	0.3945	0.074*	
H16B	0.1543	0.3870	0.3413	0.074*	
C15	0.3146 (3)	0.70842 (16)	0.22724 (9)	0.0520 (4)	
H15	0.4426	0.7229	0.1942	0.062*	
C1	0.0495 (3)	0.56932 (15)	0.08517 (9)	0.0502 (4)	
C25	0.1281 (3)	1.04863 (15)	0.08638 (10)	0.0559 (4)	
H25A	0.1144	1.1126	0.1162	0.084*	
H25B	−0.0303	1.0314	0.0742	0.084*	
H25C	0.2221	1.0741	0.0437	0.084*	
C7	0.0082 (4)	0.68946 (16)	0.03881 (10)	0.0602 (4)	
H7A	0.1240	0.6941	−0.0015	0.072*	
H7B	−0.1532	0.6962	0.0207	0.072*	
C26	0.5075 (3)	0.9593 (2)	0.14196 (11)	0.0680 (5)	
H26A	0.5898	0.8856	0.1641	0.102*	
H26B	0.5011	1.0206	0.1734	0.102*	
H26C	0.5932	0.9878	0.0983	0.102*	
C17	0.3137 (4)	0.44022 (18)	0.42382 (11)	0.0737 (6)	
H17A	0.4723	0.4420	0.3997	0.088*	
H17B	0.2929	0.5090	0.4508	0.088*	
C2	0.2552 (4)	0.4958 (2)	0.07900 (14)	0.0793 (6)	
H2	0.3759	0.5192	0.0451	0.095*	
C6	−0.1203 (4)	0.5317 (2)	0.13600 (15)	0.0838 (7)	
H6	−0.2626	0.5805	0.1410	0.101*	
C4	0.1144 (5)	0.35045 (19)	0.17350 (14)	0.0811 (7)	
H4	0.1353	0.2767	0.2032	0.097*	
C3	0.2855 (5)	0.3846 (2)	0.12373 (17)	0.0952 (8)	
H3	0.4255	0.3341	0.1188	0.114*	
C5	−0.0874 (6)	0.4237 (2)	0.18012 (16)	0.0991 (9)	
H5	−0.2059	0.4009	0.2149	0.119*	
O4	0.7030 (2)	0.29952 (12)	0.48642 (7)	0.0642 (3)	
C18	0.4982 (3)	0.26677 (14)	0.50064 (8)	0.0465 (3)	
H1N	0.159 (3)	0.3084 (18)	0.4862 (10)	0.056*	

### Atomic displacement parameters (Å^2^)

**Table:**

	*U*^11^	*U*^22^	*U*^33^	*U*^12^	*U*^13^	*U*^23^
Cl1	0.1179 (5)	0.0585 (3)	0.0845 (4)	−0.0129 (3)	−0.0086 (3)	0.0335 (3)
O3	0.0462 (6)	0.0410 (5)	0.0416 (5)	0.0000 (4)	0.0017 (4)	0.0081 (4)
O1	0.0492 (6)	0.0451 (6)	0.0538 (6)	−0.0078 (5)	−0.0060 (5)	−0.0003 (5)
C10	0.0392 (7)	0.0409 (7)	0.0330 (6)	−0.0035 (5)	−0.0046 (5)	0.0041 (5)
C19	0.0465 (7)	0.0357 (7)	0.0350 (7)	−0.0017 (6)	−0.0001 (6)	−0.0001 (5)
C11	0.0394 (7)	0.0473 (8)	0.0378 (7)	0.0005 (6)	−0.0011 (5)	−0.0013 (6)
C22	0.0610 (9)	0.0367 (7)	0.0474 (8)	0.0015 (7)	0.0019 (7)	0.0073 (6)
C13	0.0527 (8)	0.0486 (8)	0.0412 (8)	−0.0060 (7)	−0.0074 (6)	0.0104 (6)
C20	0.0461 (8)	0.0511 (9)	0.0450 (8)	−0.0073 (6)	−0.0056 (6)	0.0018 (7)
C8	0.0447 (8)	0.0491 (8)	0.0404 (8)	−0.0036 (6)	−0.0003 (6)	0.0108 (6)
C24	0.0531 (8)	0.0445 (8)	0.0447 (8)	−0.0053 (6)	−0.0119 (7)	0.0011 (6)
C12	0.0453 (8)	0.0583 (9)	0.0380 (7)	−0.0092 (7)	0.0024 (6)	0.0064 (7)
C9	0.0399 (7)	0.0464 (8)	0.0413 (7)	−0.0101 (6)	−0.0029 (6)	0.0110 (6)
C23	0.0556 (9)	0.0428 (8)	0.0548 (9)	−0.0110 (7)	−0.0031 (7)	−0.0008 (7)
N1	0.0678 (9)	0.0487 (8)	0.0573 (9)	−0.0077 (7)	−0.0119 (7)	0.0189 (7)
C14	0.0535 (9)	0.0530 (9)	0.0522 (9)	0.0118 (7)	0.0016 (7)	0.0120 (7)
O2	0.0602 (8)	0.0969 (11)	0.0716 (9)	−0.0088 (7)	0.0190 (7)	−0.0172 (8)
C21	0.0533 (9)	0.0581 (10)	0.0465 (9)	0.0024 (7)	−0.0090 (7)	0.0080 (7)
C16	0.0690 (11)	0.0530 (10)	0.0578 (10)	−0.0112 (8)	−0.0102 (8)	0.0185 (8)
C15	0.0445 (8)	0.0582 (10)	0.0451 (8)	0.0065 (7)	0.0081 (6)	0.0133 (7)
C1	0.0567 (9)	0.0442 (8)	0.0513 (9)	−0.0047 (7)	−0.0084 (7)	−0.0088 (7)
C25	0.0633 (10)	0.0434 (8)	0.0568 (10)	−0.0116 (7)	−0.0070 (8)	0.0155 (7)
C7	0.0758 (12)	0.0519 (10)	0.0539 (10)	−0.0084 (8)	−0.0143 (9)	−0.0038 (8)
C26	0.0477 (9)	0.0863 (14)	0.0692 (12)	−0.0240 (9)	−0.0087 (8)	0.0061 (10)
C17	0.1014 (16)	0.0500 (10)	0.0662 (12)	−0.0202 (10)	−0.0312 (11)	0.0239 (9)
C2	0.0739 (13)	0.0740 (14)	0.0859 (15)	0.0102 (11)	0.0138 (11)	−0.0106 (12)
C6	0.0694 (13)	0.0593 (12)	0.1124 (19)	0.0032 (10)	0.0217 (13)	0.0117 (12)
C4	0.1112 (19)	0.0473 (11)	0.0844 (16)	−0.0056 (11)	−0.0249 (14)	0.0003 (10)
C3	0.0857 (17)	0.0709 (15)	0.127 (2)	0.0317 (13)	−0.0177 (16)	−0.0201 (15)
C5	0.112 (2)	0.0640 (14)	0.111 (2)	−0.0102 (14)	0.0267 (16)	0.0167 (13)
O4	0.0664 (8)	0.0601 (8)	0.0629 (8)	−0.0201 (6)	−0.0008 (6)	0.0118 (6)
C18	0.0619 (9)	0.0400 (8)	0.0369 (7)	−0.0088 (7)	−0.0034 (6)	0.0011 (6)

### Geometric parameters (Å, °)

**Table:**

Cl1—C22	1.7359 (16)	C14—C15	1.389 (2)
O3—C10	1.3798 (16)	C14—H14	0.9300
O3—C9	1.4353 (17)	C21—H21	0.9300
O1—C8	1.3273 (19)	C16—C17	1.476 (3)
O1—C7	1.456 (2)	C16—H16A	0.9700
C10—C11	1.380 (2)	C16—H16B	0.9700
C10—C15	1.382 (2)	C15—H15	0.9300
C19—C20	1.384 (2)	C1—C6	1.358 (3)
C19—C24	1.385 (2)	C1—C2	1.360 (3)
C19—C18	1.4996 (19)	C1—C7	1.502 (2)
C11—C12	1.380 (2)	C25—H25A	0.9600
C11—H11	0.9300	C25—H25B	0.9600
C22—C21	1.369 (2)	C25—H25C	0.9600
C22—C23	1.375 (2)	C7—H7A	0.9700
C13—C14	1.378 (2)	C7—H7B	0.9700
C13—C12	1.385 (2)	C26—H26A	0.9600
C13—C16	1.516 (2)	C26—H26B	0.9600
C20—C21	1.380 (2)	C26—H26C	0.9600
C20—H20	0.9300	C17—H17A	0.9700
C8—O2	1.199 (2)	C17—H17B	0.9700
C8—C9	1.525 (2)	C2—C3	1.402 (3)
C24—C23	1.380 (2)	C2—H2	0.9300
C24—H24	0.9300	C6—C5	1.370 (3)
C12—H12	0.9300	C6—H6	0.9300
C9—C26	1.517 (2)	C4—C3	1.340 (4)
C9—C25	1.527 (2)	C4—C5	1.343 (4)
C23—H23	0.9300	C4—H4	0.9300
N1—C18	1.329 (2)	C3—H3	0.9300
N1—C17	1.462 (2)	C5—H5	0.9300
N1—H1N	0.848 (19)	O4—C18	1.224 (2)
			
C10—O3—C9	121.21 (11)	C13—C16—H16B	109.1
C8—O1—C7	117.82 (14)	H16A—C16—H16B	107.8
O3—C10—C11	115.14 (12)	C10—C15—C14	119.48 (14)
O3—C10—C15	125.57 (13)	C10—C15—H15	120.3
C11—C10—C15	119.29 (13)	C14—C15—H15	120.3
C20—C19—C24	119.15 (13)	C6—C1—C2	117.80 (19)
C20—C19—C18	117.50 (13)	C6—C1—C7	119.92 (17)
C24—C19—C18	123.33 (13)	C2—C1—C7	122.28 (18)
C10—C11—C12	120.27 (14)	C9—C25—H25A	109.5
C10—C11—H11	119.9	C9—C25—H25B	109.5
C12—C11—H11	119.9	H25A—C25—H25B	109.5
C21—C22—C23	121.71 (14)	C9—C25—H25C	109.5
C21—C22—Cl1	119.41 (13)	H25A—C25—H25C	109.5
C23—C22—Cl1	118.87 (13)	H25B—C25—H25C	109.5
C14—C13—C12	117.37 (14)	O1—C7—C1	109.73 (14)
C14—C13—C16	120.99 (15)	O1—C7—H7A	109.7
C12—C13—C16	121.64 (15)	C1—C7—H7A	109.7
C21—C20—C19	120.70 (15)	O1—C7—H7B	109.7
C21—C20—H20	119.6	C1—C7—H7B	109.7
C19—C20—H20	119.6	H7A—C7—H7B	108.2
O2—C8—O1	124.18 (17)	C9—C26—H26A	109.5
O2—C8—C9	124.20 (15)	C9—C26—H26B	109.5
O1—C8—C9	111.50 (13)	H26A—C26—H26B	109.5
C23—C24—C19	120.52 (14)	C9—C26—H26C	109.5
C23—C24—H24	119.7	H26A—C26—H26C	109.5
C19—C24—H24	119.7	H26B—C26—H26C	109.5
C11—C12—C13	121.54 (14)	N1—C17—C16	112.07 (16)
C11—C12—H12	119.2	N1—C17—H17A	109.2
C13—C12—H12	119.2	C16—C17—H17A	109.2
O3—C9—C26	112.45 (13)	N1—C17—H17B	109.2
O3—C9—C8	111.48 (11)	C16—C17—H17B	109.2
C26—C9—C8	111.72 (15)	H17A—C17—H17B	107.9
O3—C9—C25	104.40 (13)	C1—C2—C3	120.1 (2)
C26—C9—C25	109.32 (14)	C1—C2—H2	120.0
C8—C9—C25	107.05 (13)	C3—C2—H2	120.0
C22—C23—C24	118.96 (15)	C1—C6—C5	121.6 (2)
C22—C23—H23	120.5	C1—C6—H6	119.2
C24—C23—H23	120.5	C5—C6—H6	119.2
C18—N1—C17	122.04 (17)	C3—C4—C5	119.4 (2)
C18—N1—H1N	123.1 (13)	C3—C4—H4	120.3
C17—N1—H1N	114.4 (13)	C5—C4—H4	120.3
C13—C14—C15	122.03 (15)	C4—C3—C2	120.6 (2)
C13—C14—H14	119.0	C4—C3—H3	119.7
C15—C14—H14	119.0	C2—C3—H3	119.7
C22—C21—C20	118.94 (15)	C4—C5—C6	120.6 (2)
C22—C21—H21	120.5	C4—C5—H5	119.7
C20—C21—H21	120.5	C6—C5—H5	119.7
C17—C16—C13	112.54 (15)	O4—C18—N1	123.41 (15)
C17—C16—H16A	109.1	O4—C18—C19	120.39 (14)
C13—C16—H16A	109.1	N1—C18—C19	116.19 (14)
C17—C16—H16B	109.1		
			
C9—O3—C10—C11	−166.62 (13)	C23—C22—C21—C20	−0.7 (3)
C9—O3—C10—C15	13.7 (2)	Cl1—C22—C21—C20	−179.99 (13)
O3—C10—C11—C12	179.26 (13)	C19—C20—C21—C22	0.6 (3)
C15—C10—C11—C12	−1.0 (2)	C14—C13—C16—C17	70.4 (3)
C24—C19—C20—C21	0.3 (2)	C12—C13—C16—C17	−109.8 (2)
C18—C19—C20—C21	−178.30 (15)	O3—C10—C15—C14	−179.17 (15)
C7—O1—C8—O2	5.9 (2)	C11—C10—C15—C14	1.1 (2)
C7—O1—C8—C9	−178.00 (12)	C13—C14—C15—C10	−0.4 (3)
C20—C19—C24—C23	−1.1 (2)	C8—O1—C7—C1	92.40 (18)
C18—C19—C24—C23	177.38 (15)	C6—C1—C7—O1	73.1 (2)
C10—C11—C12—C13	0.1 (2)	C2—C1—C7—O1	−106.0 (2)
C14—C13—C12—C11	0.7 (2)	C18—N1—C17—C16	147.0 (2)
C16—C13—C12—C11	−179.06 (15)	C13—C16—C17—N1	176.16 (18)
C10—O3—C9—C26	−76.41 (18)	C6—C1—C2—C3	0.7 (3)
C10—O3—C9—C8	49.95 (16)	C7—C1—C2—C3	179.8 (2)
C10—O3—C9—C25	165.19 (12)	C2—C1—C6—C5	0.2 (4)
O2—C8—C9—O3	−146.77 (16)	C7—C1—C6—C5	−178.9 (2)
O1—C8—C9—O3	37.15 (16)	C5—C4—C3—C2	0.2 (4)
O2—C8—C9—C26	−20.0 (2)	C1—C2—C3—C4	−0.9 (4)
O1—C8—C9—C26	163.91 (13)	C3—C4—C5—C6	0.8 (4)
O2—C8—C9—C25	99.64 (18)	C1—C6—C5—C4	−1.0 (5)
O1—C8—C9—C25	−76.44 (15)	C17—N1—C18—O4	−1.8 (3)
C21—C22—C23—C24	−0.1 (3)	C17—N1—C18—C19	178.35 (16)
Cl1—C22—C23—C24	179.17 (13)	C20—C19—C18—O4	28.4 (2)
C19—C24—C23—C22	1.0 (3)	C24—C19—C18—O4	−150.07 (16)
C12—C13—C14—C15	−0.5 (3)	C20—C19—C18—N1	−151.74 (16)
C16—C13—C14—C15	179.19 (17)	C24—C19—C18—N1	29.8 (2)

### Hydrogen-bond geometry (Å, °)

**Table:**

*D*—H···*A*	*D*—H	H···*A*	*D*···*A*	*D*—H···*A*
N1—H1N···O4^i^	0.848 (19)	2.540 (19)	3.350 (2)	160.3 (18)
C17—H17B···O4^ii^	0.97	2.57	3.532 (3)	172.
C7—H7B···O2^i^	0.97	2.59	3.398 (3)	141.

Symmetry codes: (i) *x*−1, *y*, *z*; (ii) −*x*+1, −*y*+1, −*z*+1.

## References

[bb1] Bruker (2007). *SADABS*, *APEX2* and *SAINT* Bruker AXS Inc., Madison, Wisconsin, USA.

[bb2] Cabrero, A., Alegret, M., Sanchez, R. M., Adzet, T., Laguna, J. C. & Vazquez, M. (2001). *Diabetes*, **50**, 1883–1890. 10.2337/diabetes.50.8.1883 11473052

[bb3] Farrugia, L. J. (1999). *J. Appl. Cryst.* **32**, 837–838.

[bb4] Sheldrick, G. M. (2008). *Acta Cryst.* A**64**, 112–122. 10.1107/S0108767307043930 18156677

[bb5] Spek, A. L. (2009). *Acta Cryst.* D**65**, 148–155. 10.1107/S090744490804362X PMC2631630 19171970

